# Perception and Attitude of Parents of Children With Orofacial Clefts Regarding the Use of Presurgical Orthopedics and Feeding Obturators

**DOI:** 10.7759/cureus.46131

**Published:** 2023-09-28

**Authors:** Soha Alqadi, Ahmad Qazali, Raghad Altamimi, Rahaf Altamimi, Ismail Abdouh, Ahmad Othman, Fatma Abdulhameed

**Affiliations:** 1 Pediatric Dentistry and Orthodontics, College of Dentistry, Taibah University, Al-Madinah Al-Munawwarah, SAU; 2 Prosthodontics, College of Dentistry, Taibah University, Al-Madinah Al-Munawwarah, SAU; 3 General Dentistry, College of Dentistry, Taibah University, Al-Madinah Al-Munawwarah, SAU; 4 Oral Basic and Clinical Sciences, College of Dentistry, Taibah University, Al-Madinah Al-Munawwarah, SAU; 5 Oral and Maxillofacial Surgery, College of Dentistry, Taibah University, Al-Madinah Al-Munawwarah, SAU; 6 Pediatric Surgery, King Salman Bin Abdulaziz Medical City, Al-Madinah Al-Munawwarah, SAU

**Keywords:** cleft lip & palate, cleft lip, cleft palate, feeding obturators, presurgical nasoalveolar molding, presurgical orthopedics, orofacial clefts

## Abstract

Objective: This study aims to evaluate the parents’ attitude and their perception regarding the management of orofacial cleft (OFC) children with presurgical nasoalveolar molding (PNAM), DynaCleft and/or feeding obturators.

Material and methods: A cross-sectional, descriptive and observational retrospective survey-based study was conducted among parents of OFC children treated with PNAM, DynaCleft and/or feeding obturators who attended a primary dental health care center in Al-Madinah, Saudi Arabia, from 2019 to 2023. A validated questionnaire was used after translating it from English to Arabic. The questionnaire consisted of 32 questions divided into two sections. The first section covers parents' sociodemographic data and OFC risk factors. The second section evaluates the parents’ perception regarding the use of presurgical orthopedics (PSO) for OFC repair. The questionnaire was completed through telephone interviews carried out by two investigators with the parents of OFC children.

Results: Out of 142 parents of OFC children, only 40 parents and their children met the inclusion criteria of the study. Most parents (95%) reported their satisfaction with the treatment and stated that they would encourage other parents of OFC children to use PSO.

Conclusion: This study concluded that parents of OFC children had a positive attitude toward PSO treatment. Based on the positive outcomes of PSO treatment reported in the current study and previous literature, PSO should be considered as a routine treatment in the early management of orofacial clefts.

## Introduction

The most common craniofacial anomalies are orofacial cleft (OFC) anomalies, which are one of the most common congenital abnormalities [1]. In OFCs, the fusion of the lip and/or palate at the midline fails during development, which leads to cleft deformity in the newborn [1]. OFC can be unilateral, bilateral, complete, or incomplete and is classified as cleft lip only (CL), palate only (CPO), or both cleft lip and palate (CL/P) [2]. These features may present alone and are then called nonsyndromic cleft lip and palate (NSCL/P) or classified as syndromic when orofacial cleft features are accompanied by other signs [3]. Some of the signs that can be seen with syndromic orofacial clefts include hypodontia and characteristic facial features such as vertical maxillary excess, malar flattening and relative mandibular retrusion [4,5]. The World Health Organization (WHO) reported the prevalence of OFCs at birth and stated that OFC prevalence worldwide in the case of CL/P ranged from 3.4 to 22.9 per 10,000 births and 1.3 to 25.3 per 10,000 births for CPO [6]. In Saudi Arabia, the prevalence ranged from 0.65 to 1.9/ 1000 live births which was highest in the Al-Madinah region [7].

Extensive orofacial clefts are associated with severe nasolabial deformities which act as barriers to achieve esthetic and functional outcomes [8]. Initial treatment of orofacial clefts is provided to reduce the alveolar deformity caused by OFC through a series of treatments known as presurgical orthopedics (PSOs) [9]. In these treatments, efforts are made to bring nasolabial and maxillary segments closer to each other [9]. PSO treatment is mainly carried out in the first few weeks after birth before surgical lip and palatal repair [9]. Nonsurgical management of clefts has a long history, and various approaches were proposed to obtain an orthopedic effect [10]. In 1999, Grayson and colleagues developed presurgical nasoalveolar molding (PNAM) which combines an intraoral molding device and a nasal molding stent [11,12]. The newborn’s cartilage was observed for the early correction of congenital deformities [13]. Research revealed that the auricular cartilage is soft and plastic during the early neonatal period, and the cartilage's flexibility decreases quickly as the infant grows older. Due to the similarity between the alar and auricular cartilage. The principles for auricular cartilage correction were applied to correct cleft lip and nasal deformities [12]. In 1844, Hullihen emphasized the importance of presurgical management of clefts through the use of adhesive tape [14,15]. In 2013, the DynaCleft concept was introduced. Similar to PNAM, DynaCleft also has an alveolar and a nasal component and follows the same concept [10]. DynaCleft utilizes tapes to join the cleft segments and bring bone and tissue into a more favorable surgical position [10]. OFC is not only an esthetic concern, but it also affects functions such as speech, mastication, deglutition, and hearing [16]. Prior to palatal defect closure, newborns with OFC face various obstacles to successful feeding [17]. A feeding obturator is an appliance that creates a barrier between the nasal and oral cavities to obtain separation of the cavities [18]. It is composed of an acrylic plate inserted intraorally and placed over the hard palate closing the palatal defect. Mastication and swallowing functions are restored with the use of the feeding obturator until cleft repair [19].

In health care, the satisfaction level of patients is a significant and commonly used indicator to assess the quality of treatment [20]. The views of patients and their parents regarding these treatments are unclear. This study aims to evaluate the satisfaction level of parents and their perception regarding management of OFC children with PNAM, DynaCleft and/or feeding obturators at a primary dental care center in Al-Madinah, Saudi Arabia.

## Materials and methods

Ethical approval 

Ethical approval for the study was obtained from the Institutional Review Board (IRB), College of Dentistry, Taibah University (TUCDREC/021222/SFAlgadi) and was conducted in accordance with the guidelines of the Declaration of Helsinki (2000).

Data collection method and tools

This cross-sectional, descriptive and observational retrospective survey-based study was conducted at a primary dental care center to evaluate the perception and attitude of parents of children with OFC regarding the use of PNAM, DynaCleft and/or feeding obturators. A convenience sampling technique was used to collect the data. Parents of OFC children attending Taibah University College of Dentistry (TUCoD) dental clinics from 2019 to 2023 were invited to participate in the study. The inclusion criteria were parents of OFC children who were treated with PNAM, DynaCleft and/or feeding obturators and whom have had the appliances for no less than three months to ensure proper evaluation of the appliance effect. Parents who did not meet these criteria were not included in the study. The sample size was 142, but only 40 parents met the inclusion criteria. Data were collected using a pre-reviewed and validated questionnaire which was translated from English to Arabic. Only questions related to the aim of the current study were selected [21]. The questionnaire consisted of 32 questions divided into two sections. The first section covers parents' sociodemographic data (age, gender, education) and OFC risk factors such as consanguineous marriage, a mother with a history of medications or psychological issues and a smoker father. No data regarding mothers’ smoking were obtained due to their cultural background. The second section of the questionnaire evaluates the parents’ knowledge and perception regarding the PNAM, DynaCleft and feeding obturators for OFC repair. Two investigators were responsible for data collection through telephone interviews to fill out the questionnaire. Telephone interviews were selected as the data collection method to decrease the travel time for participants as most of them were from outside the city where the treatment was provided. In addition, it helped ensure a full understanding of the questions by the parents as opposed to a self-administrated questionnaire. Two investigators from the study team were responsible for data collection. In order to prevent intraobserver bias, all definitions and explanations regarding PSO treatment were agreed upon and standardized before initiating data collection. The principal investigator was responsible for supervising the data collection process by monitoring each investigator separately to ensure the data were collected in the same manner. After a brief explanation of the study objectives, parents were asked whether the child had PNAM, DynaCleft and/or obturators fabricated for their child. A verbal consent was then obtained by a statement of agreement from the parents. Parents were informed that all information given would be anonymous and dealt with high confidentiality.

Statistical analysis

Data were exported into Microsoft Office Excel, and descriptive analysis was carried out using the Statistical Package for Social Science 16 (SPSS, version 16, Inc, Chicago, IL, USA).

## Results

Children and parents’ demographic information

Demographic information of the OFC-affected children involved in the study is shown in Table 1. About 52.5% of the involved infants were male, and 47.5% were females. The order of the affected children within their siblings was reported to be mostly the first (20%) and second (22.5%). The majority of the families reported having only one child affected with cleft lip and palate (CLP) (87.5%). However, one family reported having three children with CLP. In Table 2, parents’ demographic information is shown. The majority of the children’s parents aged between 30 and 40 years old (50% of fathers and 55% of mothers). Regarding educational level, most fathers (40%) completed high school education, whereas 37.5% of mothers had a bachelor’s degree.

**Table 1:** Distribution of the participants according to the primary characteristics of infants diagnosed with OFC (n=40). OFC: orofacial cleft.

Characteristic	No. (%)
Gender	
Male	21 (52.5%)
Female	19 (47.5%)
Patients order among siblings	
First	8 (20.0%)
Second	9 (22.5%)
Third	7 (17.5%)
Fourth	6 (15.0%)
Fifth	5 (12.5%)
Sixth	4 (10.0%)
Seventh	1 (2.5%)
Patients order among affected	
No	35 (87.5%)
First	2 (5.0%)
Second	2 (5.0%)
Third	1 (2.5%)

**Table 2:** Distribution of the participants according to the characteristics of caregivers of OFC infants (n=40). OFC: orofacial cleft.

Characteristic	No. (%)
Father’s age (years)	
20-30	7 (17.5%)
30-40	20 (50.0%)
>40	13 (32.5%)
Mother’s age (years)	
<20	1 (2.5%)
20-30	14 (35.0%)
30-40	22 (55.0%)
>40	3 (7.5%)
Father’s education	
Primary	2 (5.0%)
Intermediate	5 (12.5%)
Diploma	2 (5.0%)
High school	16 (40.0%)
University	13 (32.5%)
Master's	2 (5.0%)
Mother’s education	
Illiterate	1 (2.5%)
Primary	5 (12.5%)
Intermediate	6 (15.0%)
Diploma	1 (2.5%)
High school	10 (25.0%)
University	15 (37.5%)
Master's	1 (2.5%)
Ph.D.	1 (2.5%)

Orofacial clefts risk factors evaluation

OFC risk factors evaluation results are presented in Table 3. Fifty-five percent of parents were in a consanguineous marriage. Planned pregnancy was reported by 70% of the parents. Eighty-five percent of mothers reported vitamins and/or folic acid intake during pregnancy. The majority of mothers reported no history of drug intake (90%), and no severe psychological issues were reported. For dietary intake, 72.5% of the mothers reported good diet consumption during pregnancy. Regarding smoking, 52.5% of fathers reported no smoking during or before pregnancy, while 47.5% of fathers reported to be smokers.

**Table 3:** Distribution of the participants according to OFC risk factors (n=40). OFC: orofacial cleft.

	Question	No No. (%)	Yes No. (%)
8	Are the parents relative?	18 (45.0%)	22 (55.0%)
9	Was the pregnancy planned?	28 (70.0%)	12 (30.0%)
10	Did the mother take vitamins and/or folic acid during this pregnancy?	6 (15.0%)	34 (85.0%)
11	Did the mother have a history of drug intake?	36 (90.0%)	4 (10.0%)
12	Did the mother have a history of severe psychological problems?	40 (100.0%)	0 (0.0%)
13	Did the mother have a good food intake?	11 (27.5%)	29 (72.5%)
14	Did the father smoke before and during pregnancy?	21 (52.5%)	19 (47.5%)

Perception and satisfaction with PNAM, DynaCleft and feeding obturators

Evaluation of parents’ perception and satisfaction with PNAM, DynaCleft and feeding obturators is shown in Table 4. Sixty percent of mothers were not worried about the impression-taking procedure, and the majority of them believed that their infants did not suffer during the procedure. Most mothers (95%) reported no signs of physical trauma following impression taking, and 85% found the procedure to be noninvasive. Regarding the clarity of instructions given by the treating dentist, most parents (82.5%) did not face difficulties in understanding and following the instructions. Eighty percent of parents agreed that OFC patients always need PSO treatment. Half of the parents did not look for information on the internet regarding PSO treatment. The majority of parents (80%) found the treatment to be useful for their child and reported improvement in their feeding following PSO. Fifty-five percent of parents reported that PSO had improved their infants’ facial appearance. For most children (80%), PSO did not have an effect on the time of the surgical correction. Eight-five percent of parents did not find visible appliances of PSO to be embarrassing. Most parents (95%) reported their satisfaction with the treatment and stated that they would encourage other parents of OFC children to use PSO. Half of the parents acknowledged that OFC had an impact on their daily life.

**Table 4:** Distribution of the participants according to parent’s perception and attitude toward PSO (n=40). OFC: orofacial cleft, PSO: presurgical orthopedics.

	Question	No No. (%)	Yes No. (%)
15	Were you worried after the explanation of the impression-taking procedure?	24 (60%)	16 (40%)
16	Do you think your infant suffered during the impression procedure?	28 (70%)	12 (30%)
17	Have you noticed any signs of intra- or extraoral physical trauma after the impression procedure?	38 (95%)	2 (5%)
18	Did you consider the impression-taking procedure invasive?	34 (85%)	6 (15%)
19	Did you face any difficulties in following the instructions of the clinician, in regard to the insertion of the feeding obturator and/or taping the elastic band?	33 (82.5%)	7 (17.5%)
20	Do you think that OFC infants always need PSO treatment?	8 (20%)	32 (80%)
21	Did you search the web looking for information about PSO treatment?	21 (52.5%)	19 (47.5%)
22	Did you find PSO treatment useful for your infant?	8 (20%)	32 (80%)
23	Did PSO improve the infant’s feeding?	7 (17.5%)	33 (82.5%)
24	Did it improve the esthetic of the infant’s facial appearance, in regard to lip, nose, and profile of the face?	18 (45%)	22 (55%)
25	Did PSO make the appointment for surgical repair earlier?	32 (80%)	8 (20%)
26	Did you find it embarrassing that your infant was wearing the appliance?	34 (85%)	6 (15%)
27	Are you satisfied with the outcomes of the PSO?	2 (5%)	38 (95%)
28	Would you encourage other parents to do the PSO treatment?	2 (5%)	38 (95%)
29	Do you think that the orthodontist is an important member of the cleft team?	4 (10%)	36 (90%)
30	Did OFC affect your family’s life?	20 (50%)	20 (50%)

Table 5 demonstrates that most of the parents (45%) noticed a positive outcome regarding the use of PNAM within the first month.

**Table 5:** Distribution of the participants according to responses to the question: when did you notice the positive correction of the cleft segments following PSO? (n=40). PSO: presurgical orthopedics.

	No. (%)
No improvement was noticed	5 (12.5%)
One month	18 (45.0%)
Two months	6 (15.0%)
Three months	4 (10.0%)
Four months	7 (17.5%)

Table 6 summarizes the number of times of appliance removal by parents for cleaning. Forty-five percent of parents removed the appliance for cleaning two times daily.

**Table 6:** Distribution of the participants according to parents’ responses to the question: how many times did you remove the appliance from your baby’s mouth for cleaning? (n=40).

Number of times of appliance removal for cleaning	No. (%)
Device was not cleaned	7 (17.5%)
1	18 (45.0%)
2	8 (20.0%)
3	3 (7.5%)
4	3 (7.5%)
8	1 (2.5%)

## Discussion

Orofacial clefts have a negative impact on the facial appearance and can also lead to impaired oral function which results in a reduced health-related quality of life [22]. Children with an orofacial cleft need lifelong multidisciplinary treatment [9]. Wide and extensive cleft lip and/or palate are usually accompanied by complex orofacial deformities, which require high efforts during the surgical treatment [10]. Presurgical orthopedics are noninvasive appliances that can reduce the severity of orofacial cleft before surgical correction [10]. These appliances facilitate primary lip, nasal and alveolar surgeries by aligning and repositioning the cleft segments and nasal cartilages to reduce the dimensions of the deformity [9,10]. This in turn improves feeding and leads to a better surgical outcome [9]. For any given treatment, patients’ satisfaction can be used to assess the treatment’s quality [19]. The parents of OFC children have an integral role in the success of OFC treatment. When reviewing the literature, only a few studies appear to have evaluated parents' views on presurgical orthopedics. The aim of this study was to evaluate the perception and satisfaction of parents of cleft lip and/or palate children regarding the use of presurgical orthopedics and feeding obturators.

The PSO treatment starts with the impression procedure. The success of the appliance is determined by the impression’s quality. Thus, the impression should be accurate and safe [23]. As this procedure could cause distress to the infant, parents were asked about their views and their infants’ attitudes toward the procedure. The majority of parents were not worried after explaining the procedure and reported no suffering from the infant during impression taking and described the procedure as noninvasive.

Eighty-two percent of parents found no problems in following the instructions for PSO insertion or taping the elastic band. However, 17% reported difficulties during the insertion procedure. Although the majority of parents understood the instructions given to them, it is important to address those who found it difficult since this difficulty could be due to inadequate explanation by the clinician on the insertion and removal method and appliance care. This reflects the importance of the clinician's role in appropriately delivering instructions and ensuring parents' understanding.

Most of the parents considered PSO treatment as a must-have treatment for all patients with CLP and agreed on its efficient effect on their infants. Eighty percent of the parents found PSO treatment useful for their infant, and 82% reported improved infant feeding using the obturators. Other studies evaluated feeding in OFC-affected infants in which infants with feeding obturators and a good parental education showed favorable outcomes and improved weight gain [24,25].

More than half of the parents were satisfied with the outcomes of the PSO treatment and showed their willingness in encouraging the treatment for other parents of OFC children. However, two of the participants reported their dissatisfaction toward the treatment and reported that their infant only wore the device once without giving a clear reason. This gives an insight into the significance of recall visits following appliance insertion to properly evaluate the appliance. In the current study sample, recall visits were not feasible for all patients since most of the patients were from different regions to the city in which treatment took place. Despite that, the majority of parents in the current study expressed their satisfaction and positive view toward PSO.

These findings were consistent with those reported in other studies. In a study done by Albustani et al., mothers of CL/CLP patients approved that PSO has improved their infants’ feeding, speech and surgical outcomes [20]. Another study reported that 70% of parents of PSO-treated infants have evaluated the treatment highly regarding feeding and were satisfied with the improvement of both feeding and esthetic outcomes [26]. Broder et al. [27] compared the postsurgical outcomes between OFC-affected infants treated with PNAM and those treated without PNAM. Parents of PNAM-treated patients reported more favorable postsurgical outcomes compared to the other group despite the more severe pretreatment condition in the PNAM-treated patients [27]. A study evaluated the response of caregivers to the early care of OFC found that caregivers of PNAM-treated children had a faster decrease in anxiety and depression with improved coping skills in comparison to caregivers whom children had no PNAM treatment [28]. The current study and the above-mentioned studies reflect the positive impact of PSO management on OFC for both parents and infants.

## Conclusions

This study concluded that parents of OFC children had a positive attitude toward PSO treatment. Treatment of OFC is multidisciplinary, and it is the clinician’s role to educate the parents of OFC children about the treatment options available. Based on the positive outcomes of PSO treatment reported in the current study and previous literature, PSO should be considered as a routine treatment in the early management of orofacial clefts.
